# Recognition and Treatment Challenges of Acute Clozapine Withdrawal Syndrome: A Case Report

**DOI:** 10.7759/cureus.25765

**Published:** 2022-06-08

**Authors:** Jared T Metropulos, Benjamin R Goldstein, Benjamin Hodapp

**Affiliations:** 1 Psychiatry and Behavioral Medicine, Medical College of Wisconsin, Milwaukee, USA; 2 Psychiatry, Wisconsin Department of Health Services, Oshkosh, USA; 3 Psychiatry and Behavioral Medicine, Medical College of Wisconsin-Green Bay, Green Bay, USA

**Keywords:** schizoaffective disorder, state psychiatric hospital, clozapine withdrawal, extrapyramidal symptoms, covid-19, acute cholinergic syndrome, clozapine

## Abstract

The limited psychiatric bedspace due to the COVID-19 pandemic and the lack of access to an up-to-date medication regimen delayed the recognition of the diagnosis and treatment for a 40-year-old man with schizoaffective disorder, bipolar type, who traveled from his home city and abruptly discontinued his prescription of clozapine. He developed a cholinergic rebound syndrome including delirium and extrapyramidal symptoms (EPS). The delay included time spent in two different medical hospitals: one awaiting psychiatric bedspace, and secondly, when the patient's cholinergic rebound syndrome was misdiagnosed as acute alcohol withdrawal. Once the etiology was recognized, he was promptly treated with anticholinergic medication (benztropine) and retitrated to his outpatient dose of clozapine leading to the resolution of symptoms including delirium and EPS. This case will discuss the challenges of continuity of care in delirious, psychotic, or otherwise confused patients, including contributions from the COVID-19 pandemic. A medication card or other improvements in medication databases that may reduce delays in treatment are discussed.

## Introduction

Clozapine withdrawal syndrome has been recognized in clinical practice for decades [1] and can include psychosis, cholinergic symptoms (nausea, vomiting, delirium), serotonergic symptoms (agitation, diaphoresis, hyperreflexia), and catatonia [2,3]. Evidence has been expanded through numerous case reports and other studies including a narrative literature review [2]. Though more likely with higher doses of clozapine, withdrawal syndromes may occur at lower doses as well [4]. Despite the increased understanding of clozapine withdrawal syndrome, the symptoms encountered are non-specific to clozapine withdrawal and may be under-recognized in clinical practice. Furthermore, the diagnosis relies on timely and accurate medication reconciliation, a ubiquitous challenge in medicine, particularly psychiatry [5].

In this case report, delayed medication reconciliation and recognition of the etiology of this patient’s presentation were exacerbated by limited resources for inpatient psychiatric treatment of COVID-19-positive individuals. As a result of delayed medication reconciliation, this patient with schizoaffective disorder, bipolar type, went over 12 days before appropriate treatment for cholinergic rebound delirium in the context of abrupt clozapine discontinuation. This case report adheres to CARE 2017 guidelines.

## Case presentation

The patient was a 40-year-old Caucasian male who was brought to a local emergency department on case day one following a concerned citizen's call to police describing the patient wandering the street and placing a variety of items (toiletries, clothing) in private mailboxes. The patient told police he had not been taking medications and needed help (he later told this author he had taken his last dose of clozapine two days prior to police contact defined as case day one). While in the emergency department (ED) on case day one, he was documented by ED staff to be grandiose and to be acting in a suspicious manner toward them. He reported vague suicidal ideation, although a suicidal plan or intent was not established. He was originally placed on a 72-hour emergency detention, although it was later discovered that the patient was already under an existing mental health commitment. In the ED, he tested positive on a rapid antigen severe acute respiratory syndrome coronavirus 2 (SARS-CoV-2) test, a urine drug screen was positive only for tricyclic antidepressants, and his blood alcohol level was less than 10 mg/dL. Valproic acid serum level on the case day one was low at 18.6 µg/mL, while a lithium serum level was negligible at <0.10 mEq/L.

The patient was refused at all psychiatric hospitals contacted due to his positive COVID-19 status with the exception of the state psychiatric hospital, although there was a waitlist for COVID-19 positive beds. As a result, the patient was instead admitted to medical hospital “A” on day one to await psychiatric placement. While at medical hospital A, he was documented to become disoriented and agitated. Psychiatry from hospital A was consulted, and the patient was started on haloperidol 5 mg twice daily, lithium 300 mg daily and 600 mg daily at bedtime, divalproex sodium delayed release 500 mg twice daily, clonazepam 0.5 mg three times daily as needed for agitation, benztropine 1 mg three times daily as needed for extrapyramidal symptoms (EPS), and quetiapine 25 mg three times daily as needed for anxiety symptoms.

A bed at the state psychiatric hospital became available, and the patient was admitted on case day four. Upon admission, he was disoriented to place and time (reporting the year as 1981), disorganized (moving linens around his room and urinating in the corner of his room), catatonic (nonverbal and staring at times), and responding to internal stimuli. A Clinical Institute Withdrawal Assessment (CIWA) was obtained with a score of 26 for nausea, emesis, tachycardia, bilateral upper and lower extremity tremors, anxiety, agitation, diaphoresis, and disorientation. The patient was then transferred to medical hospital “B” for evaluation and treatment of suspected alcohol withdrawal delirium. While at medical hospital B, the patient was not observed to be in significant alcohol withdrawal. Psychiatry from hospital B was consulted, and the patient was started on haloperidol 10 mg three times daily, benztropine 2 mg twice daily, divalproex sodium delayed release 500 mg twice daily, quetiapine 150 mg daily at bedtime, and gabapentin 300 mg three times daily. The patient remained confused, disoriented, and agitated while at hospital B.

On case day eight, the patient was transferred back to the state psychiatric hospital and was seen by the overnight and weekend provider. For unclear reasons, no medications were started. The patient had a gradual improvement in disorientation over the subsequent three-day holiday weekend. Upon examination by the primary team (authors of this case) on case day 12, behavioral disorganization and disorientation were observed, although the patient was noted to be much improved as compared to case day four documentation. Specifically, the patient demonstrated no response to internal stimuli or delusions, and he ate and slept adequately. No catatonia or EPS were observed; an abnormal involuntary movement scale (AIMS) was 0. A thorough medication reconciliation was completed, including a discussion with the patient's outpatient psychiatrist, and the following outpatient medication list was delineated: clozapine 300 mg daily at bedtime, divalproex sodium extended release 500 mg daily and 1,000 mg daily at bedtime, sertraline 100 mg daily, benztropine 0.5 mg twice daily, gabapentin 400 mg daily at 8:00 and 12:00, and 600 mg daily at bedtime, quetiapine 25 mg at 8:00, 12:00, and 16:00, and 150 mg daily at bedtime.

The patient was adamant; his alcohol use in the months prior to arrival was occasional and limited to one or two drinks per sitting. He appeared credible in denial of heavy or daily alcohol use, and this was supported by discussions with the patient’s outpatient case management team. It was also noted as supportive, but inconclusive, evidence that his blood ethanol level at the initial ED visit was <10 mg/dL and aspartate aminotransferase (AST)/alanine aminotransferase (ALT) values were normal at 31/34 U/L. Additionally, a carbohydrate-deficient transferrin (CDT) drawn later on the case day 13 was within normal limits (0.7%: reference range: 0%-1.3%). Additional substance use history included tobacco smoking (approximately one pack per day). Other substance use was denied.

Management and outcome

When the authors of this case report assumed care on day 12, the patient demonstrated improvement since his initial presentation. Accurate medication reconciliation was performed, and clozapine titration was restarted alongside benztropine 1 mg twice daily for any remaining cholinergic rebound symptoms. Benztropine was tapered as the clozapine dose was increased. Absolute neutrophil counts were monitored weekly in compliance with clozapine risk evaluation and mitigation strategy (REMS) program and were unremarkable. Divalproex sodium extended release was restarted and titrated to 1,000 mg twice daily, while gabapentin was started at 300 mg three times daily and titrated to 600 mg three times daily for anxiety.

It was expected that the patient would return to his previous baseline mental status with full orientation and improvement of psychosis and mood symptoms. The patient’s delirium did resolve, and clozapine was gradually retitrated to 225 mg daily at bedtime. Poor sleep, intermittent agitation, restlessness, and grandiosity between days 12 and 27 along with a history of poor outpatient compliance prolonged the patient’s hospitalization. Clozapine and metabolites blood level on day 27 was 198 ng/mL (obtained prior to steady state in order to verify compliance). The patient was discharged on day 29 to a step-down facility with three days of clozapine 250 mg daily at bedtime, followed by an increase to his previous outpatient dose of 300 mg daily at bedtime with scheduled outpatient psychiatry follow-up.

## Discussion

This case demonstrates the known, but underrecognized, consequences of abrupt clozapine discontinuation which were amplified by multiple care transitions due to the ongoing COVID-19 pandemic. A lack of COVID-19-positive psychiatric bedspace across the state led to an initial delay in inpatient psychiatric admission. Although psychiatry was consulted at each medical hospital, the presence of clozapine prescription was not known by hospital A as they lacked access to the patient’s up-to-date medication list. The patient was also a poor historian due to his mental status. When eventually admitted to the state psychiatric hospital, the overlap between alcohol withdrawal symptoms (nausea/vomiting, tremor, sweating, anxiety, agitation, headache, disorientation, hallucinations) and clozapine withdrawal symptoms, paired with a lack of collateral information (medication list), prompted a transfer back to a second medical hospital, hospital B. As was the case with hospital A, hospital B did not have access to the patient’s up-to-date outpatient medication list. In hindsight, the patient was suffering from cholinergic rebound delirium symptoms (disorientation, catatonia, nausea/vomiting, diarrhea, sweating, anxiety, urinary urgency), and a recent history of heavy and sustained alcohol use was not found.

It is observed that this patient’s delay in care could have been reduced with improved medication reconciliation procedures. Despite common knowledge that medication reconciliation and poor continuity of care are frequent causes of medication errors within psychiatry [6], systems often remain ineffective. During admissions to both hospitals A and B, psychiatric consultations were obtained. Again, due to the lack of an up-to-date medication list, errors were made. These included starting lithium (a prescription from years prior) and not restarting clozapine. The timing of haloperidol initiation also correlated with the onset of the patient’s tremor. Cholinergic rebound has been demonstrated to induce extrapyramidal symptoms, especially in the presence of additional D2 receptor antagonism such as haloperidol [2,3,7]. This tremor was later misidentified as an alcohol withdrawal-related symptom.

To compound the challenge of seeing psychiatric patients in the inpatient setting who are not established within the organization, there is variable sharing or access to medical records which can be referenced by providers. Psychiatric hospitals, especially on nights and weekends, may be reliant on emergency departments, pharmacies (if open), and patients who may be unable to communicate effectively. While record-sharing systems such as Epic Systems (Verona, Wisconsin, United States) “Care Everywhere” have improved inter-organizational communication [8], problems remain. Within the mental health system, mental health records may be kept private, or the patient may utilize outside care such as county mental health services which may keep records on alternative systems. While systems and organizations continue to work toward enhanced record-sharing capabilities, they should also consider resources to assist patients in carrying a medication list, especially those prescribed medications which could lead to harm if not continued during a care transition or emergency. Medication cards, medical bracelets, or other forms of medical ID could also be considered to aid in the recognition of psychiatric conditions or specific medications [9]. The authors suggest consideration for expansion of existing registries, such as the Prescription Drug Monitoring Program (PDMP) or the Clozapine Risk Evaluation and Mitigation Strategy (REMS) given the risks of clozapine including abrupt discontinuation.

It is recalled that the patient’s lack of adherence to clozapine led to the events as described in this case. Adherence issues are a well-known challenge in medicine including psychiatric patients [10]. While clozapine adherence rates are comparable to other oral antipsychotics [11], the overall rate of adherence to oral antipsychotics in patients with schizophrenia is in the range of 70% [12]. As a result, options to improve adherence have been developed such as liquid or orally disintegrating tablets (ODTs), long-acting injectable formulations [13], and therapeutic drug monitoring via antipsychotic serum levels. Clozapine does have liquid and ODT formulations available, although it lacks a long-acting injectable option. The serum level of clozapine can be drawn and processed by some treatment centers, but many facilities (including the state psychiatric hospital in this case) rely on a send-out lab for clozapine. This can take a week or more to get the result, limiting the clinical utility of such a strategy.

Finally, this case provides an opportunity to review the best practice management of cholinergic rebound. Anticholinergic medication is effective at both treating and preventing cholinergic rebound [14]. Anticholinergic medication should generally be tapered over several weeks, although specific timing and strategies will depend on the nature of each individual case. Anticholinergic equivalents are understood as follows [3]: in tobacco smokers, benztropine 1 mg = trihexyphenidyl 2.5 mg= diphenhydramine 25 mg = clozapine 100 mg; and in nonsmokers, benztropine 1 mg = trihexyphenidyl 2.5 mg = diphenhydramine 25 mg = clozapine 50 mg.

## Conclusions

This case demonstrates the underrecognized clozapine withdrawal syndrome while highlighting many of the clinical challenges which can arise related to the prescribing of clozapine. Altered mental status, psychiatric bedspace limitations during the COVID-19 pandemic, and lack of adequate medication reconciliation all contributed to the complications described in this case. The diagnosis of cholinergic rebound delirium was also initially overlooked due to the nonspecific symptoms overlapping with the much more common alcohol withdrawal delirium. The astute clinician will maintain clozapine withdrawal on their differential during the evaluation of psychosis, altered mental status, catatonia, and cholinergic syndromes. Once cholinergic rebound in the context of abrupt or rapid clozapine discontinuation is diagnosed, patients should be treated with anticholinergic agents (benztropine, trihexyphenidyl, diphenhydramine). In cases where clozapine discontinuation is performed under the care of a medical professional, cholinergic rebound syndrome can be prevented by a gradual clozapine taper. This taper may include starting additional anticholinergic medications as determined by the clinical scenario. 

This case provides a compelling reason for the consideration of a more widespread medication registry, which would be of benefit in cases of delirium or altered mental status, particularly when a patient is being treated at an organization that does not generally provide their care. Improved organizational record sharing and/or the encouragement of patients to utilize a medication card or wear a medical alert bracelet could also be considered. 
